# Magnetic resonance imaging findings of cystic ovarian tumors: major differential diagnoses in five types frequently encountered in daily clinical practice

**DOI:** 10.1007/s11604-022-01321-x

**Published:** 2022-08-02

**Authors:** Ayumi Ohya, Yasunari Fujinaga

**Affiliations:** Department of Radiology, Shinshu University School of Medicine, 3-1-1 Asahi, Matsumoto, 390-8621 Japan

**Keywords:** Cystic ovarian tumor, Differential diagnosis, Magnetic resonance imaging

## Abstract

There are many types of ovarian tumors, and these different types often form cystic masses with a similar appearance, which can make their differentiation difficult. However, with the exclusion of rare ovarian tumors, the number of ovarian tumors encountered in daily practice is somewhat fixed. It goes without saying that magnetic resonance imaging (MRI) is useful for differentiating ovarian tumors. In this review, we summarize the differential diagnoses for each of the five types of MRI findings commonly encountered in daily practice. First, unilocular cystic masses without mural nodules/solid components include benign lesions such as serous cystadenoma, functional cysts, surface epithelial inclusion cysts, paratubal cysts, and endometriosis. Second, multilocular cystic ovarian lesions include mucinous tumors and ovarian metastases. It should be noted that mucinous tumors may be diagnosed as borderline or carcinoma, even if no solid component is observed. Third, cystic lesions with mural nodules that are unrelated to endometriosis include serous borderline tumor and serous carcinoma. Cystic lesions with solid components are more likely to be malignant, but some may be diagnosed as benign. Fourth, ovarian tumors deriving from endometriosis include seromucinous borderline tumors, endometrioid carcinoma, and clear cell carcinoma. These tumors sometimes need to be differentiated from serous tumors. Finally, cystic lesions with lipid contents include teratoma-related tumors. In mature cystic teratoma, mural nodules (called “Rokitansky protuberance” or “dermoid nipple”) are sometimes seen, but they do not suggest malignancy. Some of these lesions can be diagnosed accurately by considering their characteristic imaging findings, their changes over time, MRI findings other than those of the primary lesion, and information from other modalities such as tumor markers. To ensure the optimal treatment for ovarian tumors, it is important to estimate the histological type as well as to diagnose whether a lesion is benign or malignant.

## Introduction

There are numerous histological types of ovarian tumors; the WHO classification of tumors 5th edition lists approximately 70 histological types [1]. The large number of histological types means that it can be difficult to be familiar with the imaging findings of all types of ovarian tumors. Furthermore, the imaging findings of lesions that occur in sites or organs other than the ovary are often similar to those of ovarian tumors, and these tumors should be differentiated from ovarian tumors. The imaging diagnosis of ovarian tumors, therefore, requires a great deal of experience and knowledge.

However, differentiating between different types of cystic solid lesions provides important clues in the differentiation of ovarian tumors. Epithelial tumors including serous tumors, mucinous tumors, endometrioid tumors, and clear cell tumors, as well as germ cell tumors including mature teratomas, are often seen as cystic masses [1]. In contrast, the sex-cord stromal tumors of fibroma and thecoma, and germ cell tumors including dysgerminoma, are typical ovarian tumors seen as solid masses [1]. However, histologically solid ovarian tumors are sometimes observed as cyst-predominant lesions, and often need to be differentiated from cystic ovarian tumors. In addition, non-neoplastic lesions such as endometriotic cysts (both inside and outside the ovary), paratubal cysts, and peritoneal inclusion cysts are also differential diagnoses for cystic ovarian tumors. It is clinically important to be aware that some epithelial tumors such as endometrioid carcinoma and clear cell carcinoma originate from endometriosis [2]. In general, ovarian tumors composed only of cystic components without solid components are benign, whereas cystic ovarian tumors with solid components are borderline malignant or malignant [3].

In this review, we describe ovarian tumors observed as cystic masses. We focus on ovarian tumors that are or are mostly cystic in their overall composition. We classify these tumors into five types and list their differential diagnoses that are commonly encountered in daily clinical practice in Table 1. The five types of tumors are: unilocular cystic mass without solid component; multilocular cystic mass; cystic mass with mural nodule or solid component unrelated to endometriosis; cystic mass with mural nodule or solid component related to endometriosis; cystic mass with a fat or lipid content. Non-neoplastic cystic lesions that should be differentiated from ovarian tumors of each type are also described.

**Table 1:** Differential diagnoses of 5 lesion appearances

A: Unilocular cystic mass without solid component	B: Multilocular cystic mass	C: Cystic mass with solid component unrelated to endometriosis	D: Cystic mass with solid component related to endometriosis	E: Cystic mass with a fat or lipid content
Serous cystadenoma^a^	Mucinous tumors	Serous borderline tumor	Seromucinous borderline tumor	Mature cystic teratoma
Surface epithelial inclusion cyst	Ovarian metastasis	High-grade serous carcinoma	Endometrioid carcinoma	Malignant transformation of mature cystic teratoma
Functional cyst	Struma ovarii	Fibroma	Clear cell carcinoma	
Paratubal cyst	Granulosa cell tumor	Thecoma	Polypoid endometriosis	Mucinous tumors arising in mature cystic teratoma
Hydrosalpinx	Hyperreactio luminalis	Benign Brenner tumor	Decidualized endometriosis	
Endometriotic cyst^a^	Peritoneal inclusion cyst	Adenofibroma		

^a^Serous cystadenoma and endometriotic cyst may occasionally be multilocular cystic masses

## Unilocular cystic mass without solid component

Simple cystic masses are a frequently encountered pattern of ovarian mass in clinical practice. According to the O-RADS MRI Risk Stratification System, unilocular cystic lesions without solid tissue are scored as 2 or 3, regardless of their content, with a positive predictive value for malignancy of 5% or less [4, 5]. Moreover, simple cysts smaller than 3 cm seen in premenopausal women are considered functional cysts, with a score of 1. In addition, extraovarian unilocular cystic lesions without solid tissue, such as hydrosalpinx and paratubal cysts, should also be considered.

### Serous cystadenoma

**Fig. 1:**
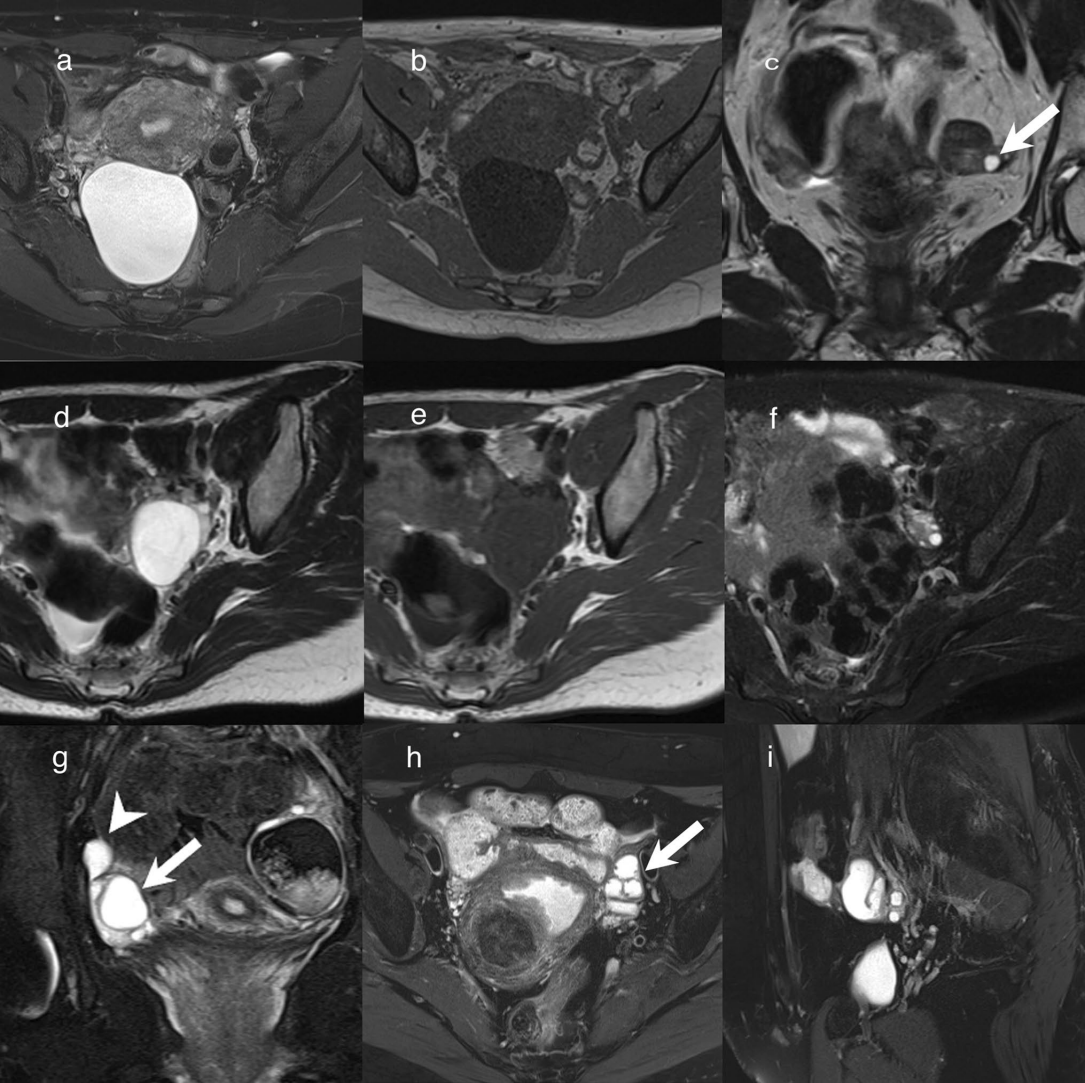
Lesions that need to be differentiated from serous cystadenoma. **a**, **b** Unilateral cystic mass of the right ovary in a woman in her 40 s. The inside of this thin-walled cystic lesion is hyperintense on T2WI (**a**) and hypointense on T1WI (**b**). There is no mural nodule inside the cystic lesion. The lesion was diagnosed as serous cyst adenoma. **c**: Unilateral small cyst (< 10 mm) (arrow) of the left ovary in a woman in her 80 s. The inside of this cystic lesion is markedly hyperintense on T2WI. The lesion was diagnosed as surface epithelial inclusion cyst. **d**–**f** Unilateral cystic mass of the left ovary in a woman in her 30 s. The inside of this thin-walled cystic lesion is markedly hyperintense on T2WI (**d**) and hypointense on T1WI (**e**). There is no mural nodule inside the cystic lesion. It could not be identified on fat-suppressed T2WI (FS-T2WI) after 3 months, and was considered to be a functional cyst (**f**). **g** Right paratubal cyst in an adolescent girl. FS-T2WI shows an extraovarian unilateral cystic lesion (arrowhead) bordering the right ovary (arrow). **h**, **i** Hydrosalpinx in a woman in her 50 s. T2WI shows a cystic lesion with marked hyperintensity on the left side of the uterus (arrow) (**h**). Linear structures showing plica of the fallopian tubes are visible inside (**h**). Sagittal FS-T2WI shows the cystic lesion is a tubular lesion with a C-shaped appearance (**i**)

Serous cystadenoma is a common form of benign serous tumor. Two-thirds of benign ovarian epithelial tumors are benign serous tumors [6], and the majority of serous tumors are benign. Serous cystadenomas can be up to 30 cm in size, but average 5–8 cm [6]. Bilateral serous cystadenoma is found in 12–23% of cases [6]. Microscopically, the epithelial lining of serous cystadenoma consists of non-layered columnar cells resembling tubal ciliated cells [1, 6]. Serous cystadenoma is filled with clear watery fluid [1, 6], and on MRI it is typically seen as a unilocular cystic mass with a thin smooth wall (Fig. 1a, b) [3, 7]. The cyst is markedly hyperintense on T2-weighted imaging (T2WI) and hypointense on T1-weighted imaging (T1WI; Fig. 1a, b). However, the contents of the cyst may not have the same MRI intensity as water because of hematological or protein-rich components. In addition, serous cystadenoma is sometimes seen as a multilocular cystic mass.

### Surface epithelial inclusion cyst

Surface epithelial inclusion cysts are often found on microscopical examination, but are sometimes found in the ovaries of postmenopausal females on MRI [8]. They are considered to be one of the origins of ovarian epithelial tumors, and can be recognized as a unilocular cystic structure less than 1 cm [9]. On MRI, the inside of the cyst is usually hypointense on T1WI and hyperintense on T2WI (Fig. 1c). Microscopically, surface epithelial inclusion cysts are composed of a single layer of epithelial cells and resemble tubal ciliated cells [9]. Therefore, surface epithelial inclusion cyst and serous cystadenoma have the same histopathological features, and the only difference is their size. If a tumor is more than 1 cm, it is diagnosed as serous cystadenoma. And surface epithelial inclusion cysts should be differentiated from follicle cysts on MRI. Follicle cysts are usually not observed after menopause [8], but it should be note that some follicle cysts may be present even after menopause [10].

### Functional cyst

Both follicle cysts and corpus luteum cysts are included in the description ‘functional cysts’. Functional cysts are menstrual cycle-related ovarian retention cysts found at reproductive age. They have a unilocular cystic structure with no internal septa or mural nodules. Large functional cysts reach 3–8 cm in size [1]. On MRI, functional cysts are markedly hyperintense on T2WI and hypointense on T1WI, but sometimes show as hyperintense on T1WI because of hematological components (Fig. 1d–f). In addition, the cyst wall sometimes shows as strongly hyperintense on contrast-enhanced fat-suppressed T1WI. The imaging features of functional cysts are similar to those of serous cystadenomas and sometimes endometriotic cysts. However, functional cysts change with the menstrual cycle and disappear after a maximum of 2 months (Fig. 1f) [11]. Therefore, if a functional cyst is suspected, it is important to ensure follow-up with ultrasonography 2 months later.

### Paratubal cyst

Paratubal cysts are cystic lesions located between the ovary and fallopian tube. They are often lined by ciliated epithelium and are classified into three types: paramesonephric, mesothelial, and mesonephric types [12]. The size of paratubal cysts varies. On MRI, they have a thin wall and appear as a well-defined unilocular cystic mass with marked hyperintensity on T2WI and hypointensity on T1WI (Fig. 1g) [11]. Although their MRI findings are very similar to those of serous cystadenoma of the ovary, paratubal cysts occur outside the ovary and can be diagnosed if they are located outside the ovary on MRI [11]. Observation with multiple cross sections is helpful for the diagnosis of paratubal cysts.

### Hydrosalpinx

The normal fallopian tube has a length of approximately 10 cm, but it cannot be identified on MRI unless liquid is present inside it. Hydrosalpinx is caused by adhesion and obstruction of the fallopian tubes due to endometriosis and salpingitis. Hydrosalpinx is described as being sausage-like or C- or S-shaped on MRI (Fig. 1h, i) [11]. If the cyst’s components are close to water, the cyst shows marked hyperintensity on T2WI and hypointensity on T1WI. Linear structures showing the plica of the fallopian tubes are often found inside (Fig. 1h) [13]. The content may be blood or abscess. In the former case, the lumen is often hyperintense on fat-suppressed T1WI, whereas in the latter case, the lumen is hyperintense on diffusion-weighted imaging (DWI) and hypointense on apparent diffusion coefficient (ADC) maps [14].

### Endometriotic cyst

**Fig. 2:**
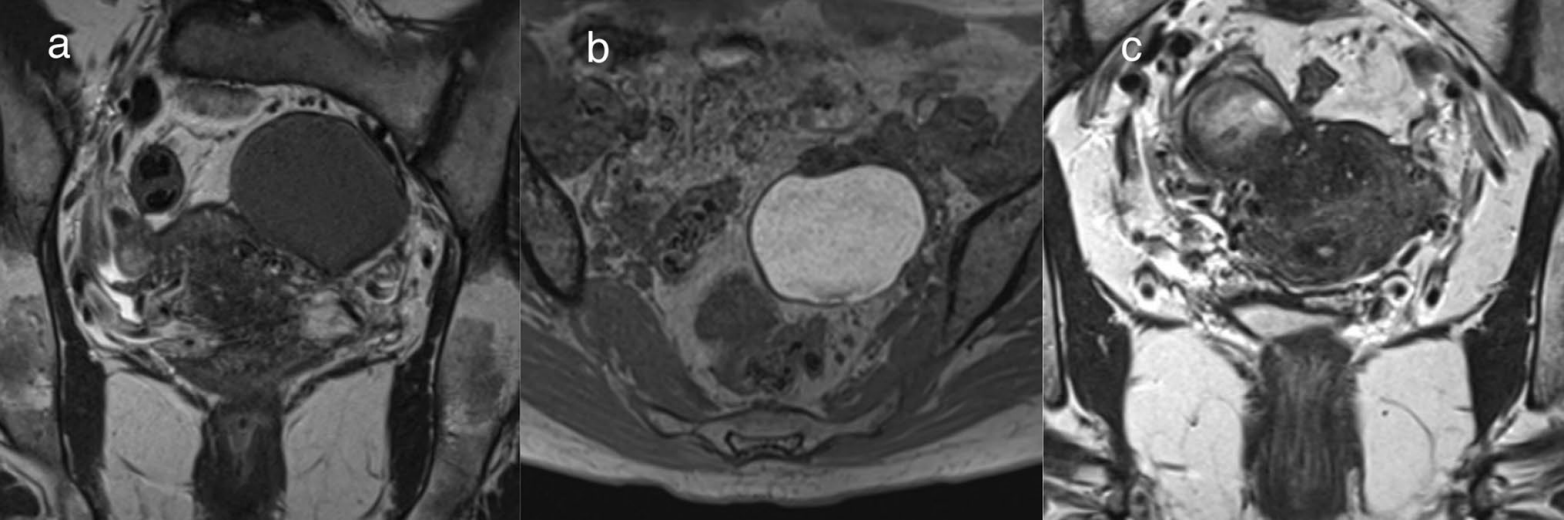
Endometriotic cyst. **a**, **b** Endometriotic cyst of the left ovary in a woman in her 40 s. It has a smooth wall and the inside of the cyst shows hypointense on coronal T2WI (**a**). The inside intensity of the cyst is higher than that of fat on T1WI (**b**). **c** Endometriotic cyst of the right ovary in a woman in her 40 s. The coronal T2WI shows a cystic lesion in contact with the uterus and a hypointense area in the myometrium at the site of adhesion, suggestive of adenomyosis

Endometriotic cysts arise from ectopic endometrial glands outside the uterus. It is said that approximately 10% of women of reproductive age have endometriosis in the pelvis [2]. The most common organ to show endometriosis is the ovary (17–65%), followed by uterine ligaments (3–69%) such as the uterosacral ligament and broad ligament, fallopian tubes (10–44%), and peritoneum (6.4–15.2%) [2]. On MRI, endometriotic cysts are observed as a unilocular or multilocular cystic mass (Fig. 2). The intensity of endometriotic cysts show equal to or higher than that of fat on T1WI. Endometriotic cysts show as hypointense to hyperintense on T2WI (Fig. 2) [15]. Occasionally, ‘shading’ on T2WI is seen as a finding of endometriotic cysts [14]. If the endometriotic lesion is in contact with the uterus, adenomyotic lesions that infiltrate the myometrium of the uterus from the contact site may be present (Fig. 2). It should be noted that some tumors such as endometrioid carcinoma, clear cell carcinoma, and seromucinous borderline tumor originate from endometriotic lesions [2]. In these tumors, solid components with a contrast-enhancement effect are found on MRI [16], although there is no solid component in most cases of endometriotic cysts.

## Multilocular cystic masses

In this section, we describe lesions composed of multilocular cystic masses with or without solid components. The score for this type of lesion ranges from 3 to 5 according to O-RADS MRI [4, 5]. Mucinous tumors are one of the most common ovarian multilocular cystic tumors, and a malignancy classification is also described for them. Hyperreactio luteinalis and peritoneal inclusion cyst are also known as multilocular cystic lesions.

### Primary ovarian mucinous tumors

**Fig. 3:**
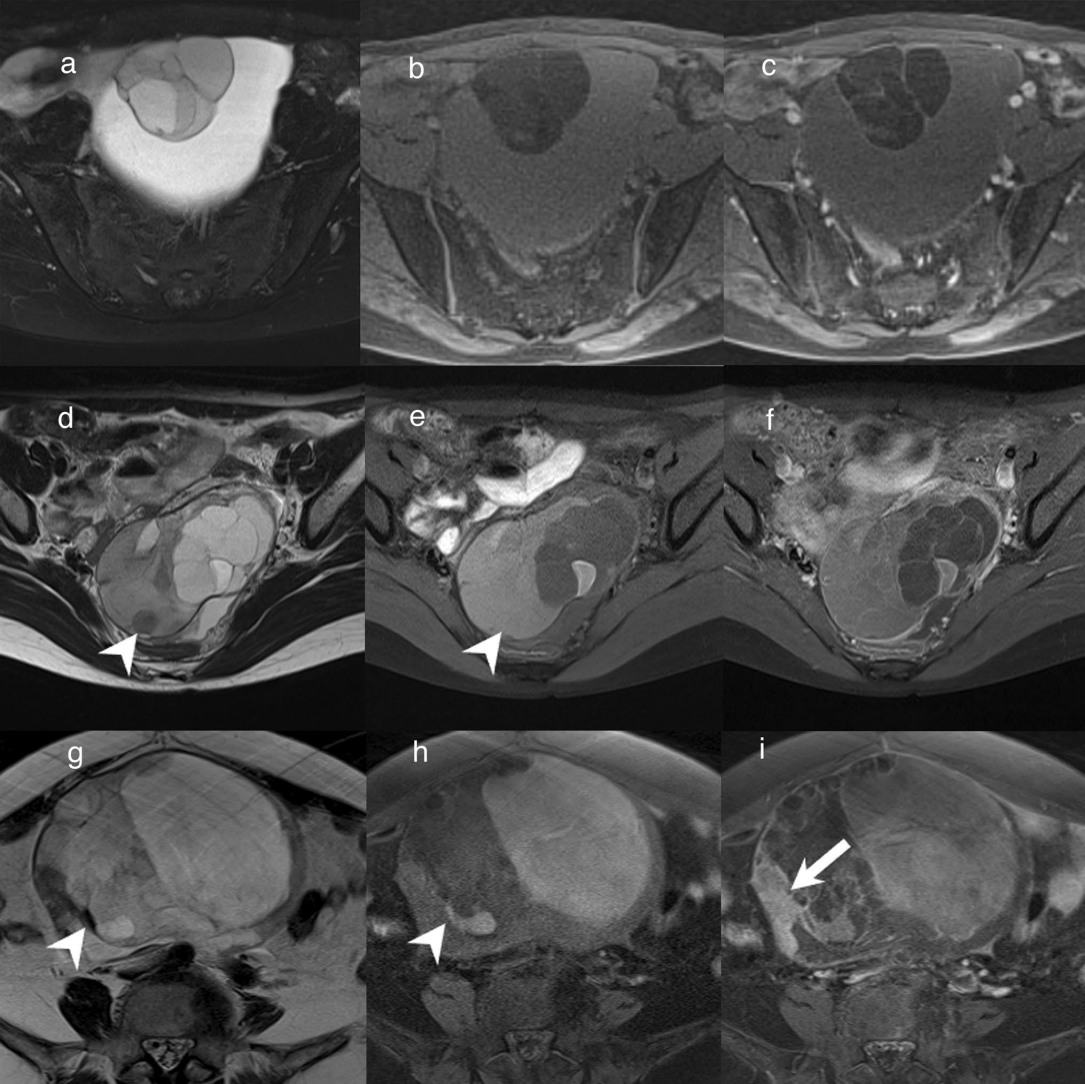
Mucinous tumors. **a**–**c** Mucinous cystadenoma of a woman in her 30 s. A multifocal cystic mass greater than 10 cm is observable in the pelvis. On FS-T2WI and fat-suppressed T1WI (FS-T1WI), the signal intensity varies across the different loculi of the mass, giving it a stained-glass appearance (**a**, **b**). Gd-enhanced FS-T1WI shows no solid components in the mass (**c**). **d**–**f** Mucinous borderline tumor in a woman in her 20 s. T2WI and FS-T1WI show different signal intensities across the loculi of the mass (**d**, **e**). The sizes of the loculi are smaller than in mucinous cystadenoma, and they are more numerous. There are loculi that are hypointense on T2WI and hyperintense on FS-T1WI (arrowhead) (**d**, **e**). Gd-enhanced FS-T1WI shows no solid components in the mass (**f**). **g**–**i** Mucinous carcinoma of a woman in her 60 s. T2WI and FS-T1WI show variations in signal intensity across the loculi of the mass (**g**, **h**). The loculi are as small as in mucinous borderline tumor. There are loculi that are hypointense on T2WI and hyperintense on FS-T1WI (arrowhead) (**g**, **h**). Gd-enhanced FS-T1WI shows enhanced solid components in the mass (arrow) (**i**)

Primary ovarian mucinous tumors are neoplasms composed of gastrointestinal-type cells [1, 6]. Primary ovarian mucinous tumors have three malignancy grades: benign, borderline malignant (borderline), and malignant. In mucinous carcinoma, the carcinoma part, benign parts with cell atypia, and borderline parts often coexist [6]. Therefore, primary ovarian mucinous tumors have been considered to exhibit an adenoma–borderline–carcinoma sequence [6]. Primary ovarian mucinous tumors are typically observed as large unilateral multiloculated lesions with a smooth outer surface and mucinous contents [1, 6]. MRI findings reflect these macroscopic features (Fig. 3) [7, 11]. Each loculus is separated by many septa, and the signal intensity of the loculi varies; these findings create what is referred to as a ‘stained-glass appearance’ (Fig. 3) [17]. Loculi tend to be more numerous and smaller in mucinous borderline tumor than in mucinous cystadenoma (Fig. 3) [18]. The imaging findings of mucinous carcinoma are typically those of mucinous borderline tumor plus solid components (Fig. 3). However, a recent study found similar numbers of loculi in mucinous borderline tumor, mucinous carcinoma, and mucinous cystadenoma, with 50% of mucinous carcinoma lacking solid components [19]. Mucinous borderline tumor and mucinous carcinoma without a solid component have an O-RADS MRI score of 3, and might be underestimated as a benign lesion [4, 5]. Recent study has focused on the intensity of cyst contents in addition to the number of loculi and solid components when grading mucinous tumors on MRI [19]. It was reported that a combination of hyperintense loculi on T1WI and hypointense loculi on T2WI suggests a high possibility of borderline or malignant mucinous tumor (Fig. 3) [19].

### Ovarian metastases from colorectal cancer

**Fig. 4:**
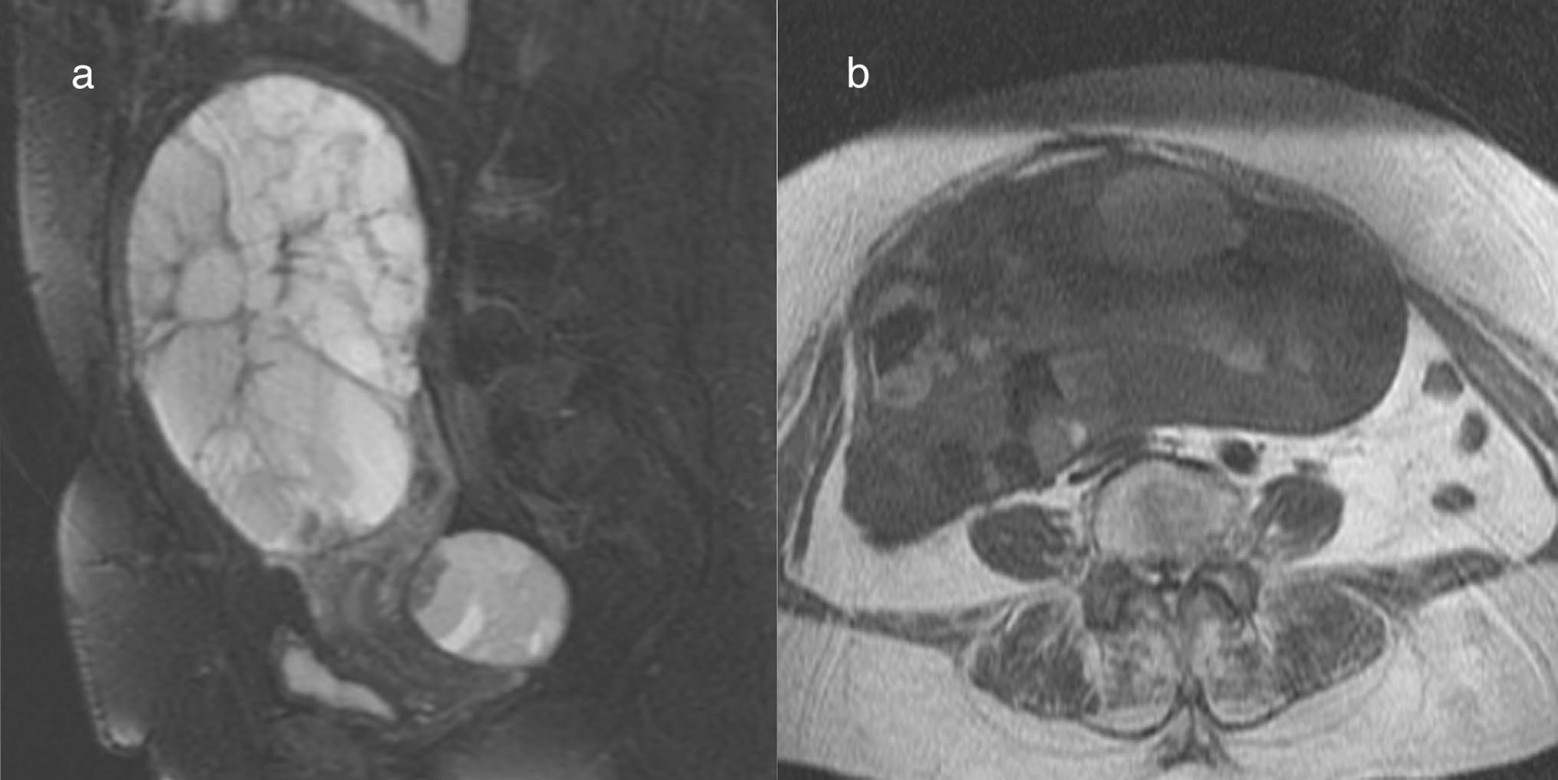
Bilateral ovarian metastasis from colon cancer in a woman in her 70 s. **a** Two multilocular cystic masses are seen at the head of the uterus and in the Douglas fossa on FS-T2WI. **b** The T1WI shows variation in the signal intensity of the loculi inside the mass, giving a ‘stained-glass’ appearance

Ovarian metastases account for 3–30% of malignant ovarian tumors [1], with the colon being the most frequent site of origin [1]. The MRI findings of ovarian metastases depend on the primary site; those from gastric cancer and breast cancer often appear as solid masses [20], whereas those from colorectal cancer appear as multilocular cystic masses and should be differentiated from primary ovarian mucinous tumors (Fig. 4) [20]. Although the imaging findings of ovarian metastases are very similar to those of primary ovarian mucinous tumors, most primary ovarian mucinous tumors are unilateral, whereas metastases from colorectal cancer are often bilateral (Fig. 3) [1, 20]. In addition, ovarian metastases from colorectal cancer have been reported to be smaller in size than primary ovarian mucinous tumors [21]. However, it should be noted in the imaging report that lower gastrointestinal endoscopy is mandatory when a unilateral smaller multilocular stained-glass-like cystic mass is encountered, because some ovarian metastases may show as unilateral lesion.

### Struma ovarii

**Fig. 5:**
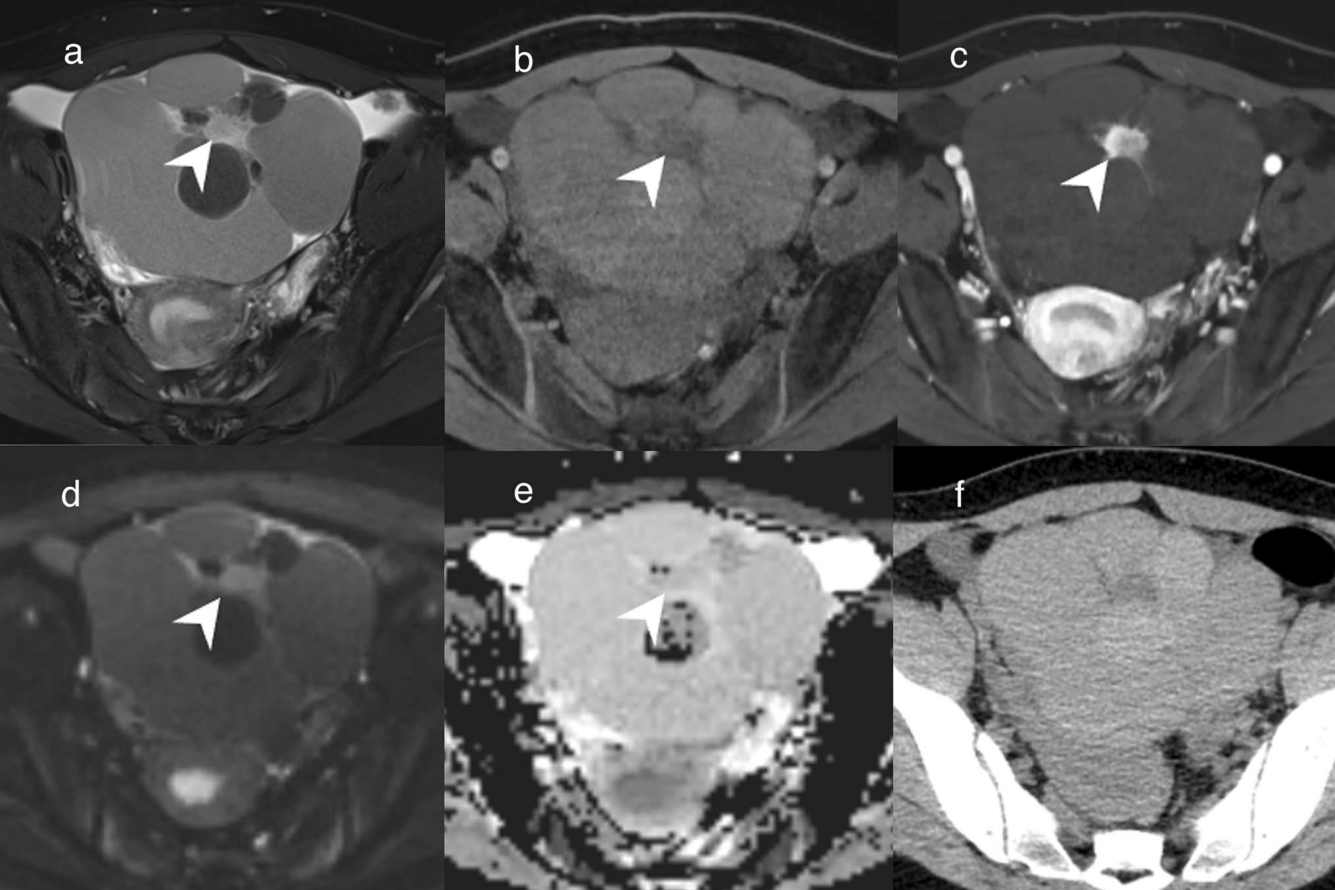
Struma ovarii in an adolescent girl. **a** FS-T2WI shows a lobulated multilocular cystic mass in the pelvis. Some loculi are as hypointense as skeletal muscle. A nodular area with hyperintensity is observable in the center of the mass (arrowhead). **b** On FS-T1WI, the majority of the mass is slightly hyperintense compared with skeletal muscle. A nodular area in the center of the mass is hypointense (arrowhead). **c** Gd-enhanced FS-T1WI shows no enhancement in the majority of the mass, but marked enhancement in the central nodular area (arrowhead). **d** The central nodular area is hyperintense on DWI (arrowhead) and ADC maps (**e**; arrowhead); hence, the area has no diffusion restriction. **f** Unenhanced CT shows the majority of the mass to be hyperdense compared with skeletal muscle, with the exception of the central nodular area

Struma ovarii is a type of monodermal teratoma that in almost all cases is composed of thyroid tissue [1]. Struma ovarii is the most common of the monodermal teratomas [1]. On MRI, it often appears as a lobular multilocular cystic mass with a stained-glass appearance, as in primary ovarian mucinous tumors (Fig. 5) [22]. Solid components of various sizes coexist in the tumor [22], and struma ovarii without solid components is rare [1]. On T2WI, some cysts show as a hypointense area reflecting viscous colloid (Fig. 5) [22]. It is reported that marked enhancement of the solid components is seen without diffusion restriction. However, struma ovarii needs to be differentiated from mucinous carcinoma because it has a solid component (Fig. 5) [22]. Fat intensities indicating teratoma are occasionally observed, and these are a key to diagnosing struma ovarii.

### Adult granulosa cell tumor

**Fig. 6:**
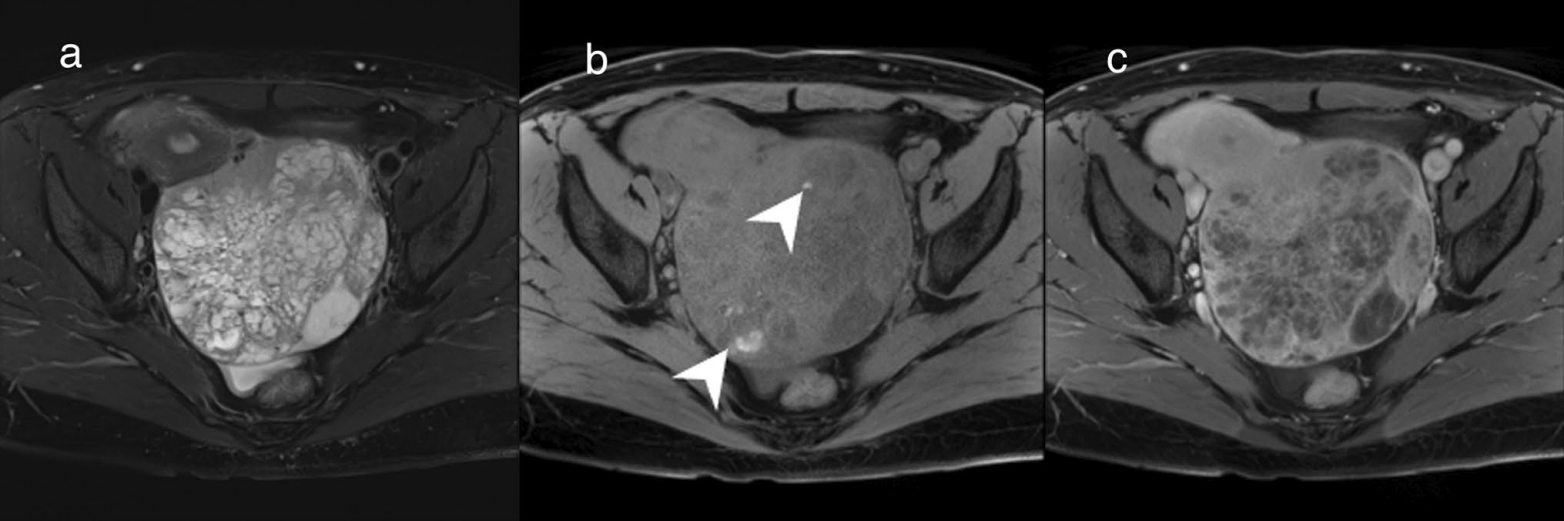
Adult granulosa cell tumor in a woman in her 40 s. **a** An internal heterogeneous mass in the left ovary appears spongy on FS-T2WI with marked hyperintense areas clustered in the center of the mass. **b** FS-T1WI shows hyperintense areas suggestive of hemorrhage (arrowhead). **c** The margins and septa of the mass show enhancement on Gd-enhanced FS-T1WI

Granulosa cell tumor is the most common of the sex-cord-stromal tumors of the ovary [1]. It accounts for 1% of all ovarian tumors, and is classified into adult (95%) and juvenile (5%) types [1, 23]. Macroscopically, adult type is a unilateral mass with an average diameter of 10 cm and typically consists of solid and cystic components [1]. Intratumor hemorrhage is common [1]. A variety of architectural patterns can be seen, and cystic structures of various sizes can be confirmed when a macrofollicular pattern is exhibited [1]. On MRI it is usually seen as a mass consisting of multilocular solid and cystic components with hemorrhage, reflecting its architectural properties (Fig. 6) [24]. It also appears as a multilocular mass composed of predominantly cystic components, which is relatively characteristic of it being a sponge-like mass (Fig. 6) [24]. Hyperintense areas on fat-suppressed T1WI indicating hemorrhage may be observed in either the solid or cystic components. Adult granulosa cell tumor also produces estrogen [1]. In postmenopausal women, estrogenic effects such as endometrial thickening and complications of endometrial carcinoma may be seen as secondary findings [25].

### Hyperreactio luminalis

**Fig. 7:**
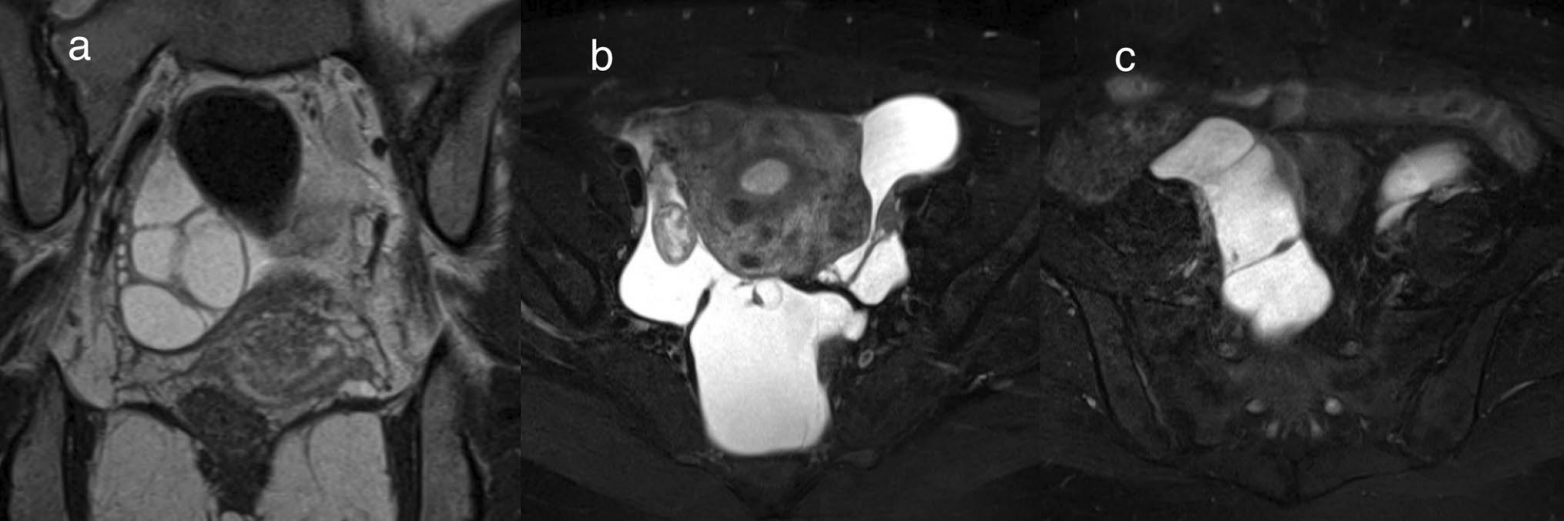
Non-neoplastic lesion presenting as a multilocular cystic mass. **a** Hyperreactio luminalis in a woman in her 30 s at 14 weeks of pregnancy. The right ovary is enlarged and a multilocular cystic mass can be seen on T2WI. Each loculus appears to be an overly-enlarged normal follicle. **b**, **c** Peritoneal inclusion cyst after uterine myomectomy in a woman in her 40 s. A cystic mass with septa can be seen along the right pelvic wall on FS-T2WI (**b**). The bilateral ovaries are inside the cystic lesion on FS-T2WI. The periphery of the cystic lesion is in line with the peritoneum (**c**)

Hyperreactio luminalis is an ovarian swelling caused by a high human chorionic gonadotropin level [1]. It occurs with pregnancy and gestational trophoblastic diseases [1]. Histopathologically, the development of many follicles and a high degree of luteinization are observed [1]. On MRI, many enlarged follicles appear as a multilocular cystic mass (Fig. 7a) [26], usually occurring in the bilateral ovaries [1]. Occasionally, there is hemorrhage in the cystic components. Mucinous tumor is an important differential diagnosis. It is important to follow-up the lesion using ultrasonography if hyperreactio luminalis is suspected during pregnancy. In a patient with hyperreactio luminalis, the cystic component will shrink or disappear during pregnancy.

### Peritoneal inclusion cyst

Peritoneal inclusion cyst, which sometimes looks like a cystic lesion, is an accumulation of ascites due to adhesions of the peritoneum and decreased absorption of ascites by the peritoneum; it is caused by surgery, pelvic infection, or endometriosis [27]. The lumen of the peritoneal inclusion cyst may be communicated, or it may be septate and seen as a multilocular lesion (Fig. 7b, c) [27, 28]. The tips for differentiating peritoneal inclusion cyst from tumors presenting as multilocular cystic masses are its irregular shape with filling of the pelvic cavity and a normal ovary inside the lesion (Fig. 7c) [29].

## Cystic mass with mural nodule or solid component unrelated to endometriosis

When solid components are observed in an ovarian cystic mass, it is likely to be a borderline or malignant tumor [3]. Many such tumors are scored as 4 or 5 on the O-RADS MRI Risk Stratification and Management System [4]. However, there is an exception when the solid components have hypointensity on T2WI and DWI, in which case it scores 2, indicating a high likelihood of benignity [4]. The differential diagnoses for a cystic mass with solid components that is not associated with endometriosis are described below.

### Serous borderline tumor

**Fig. 8:**
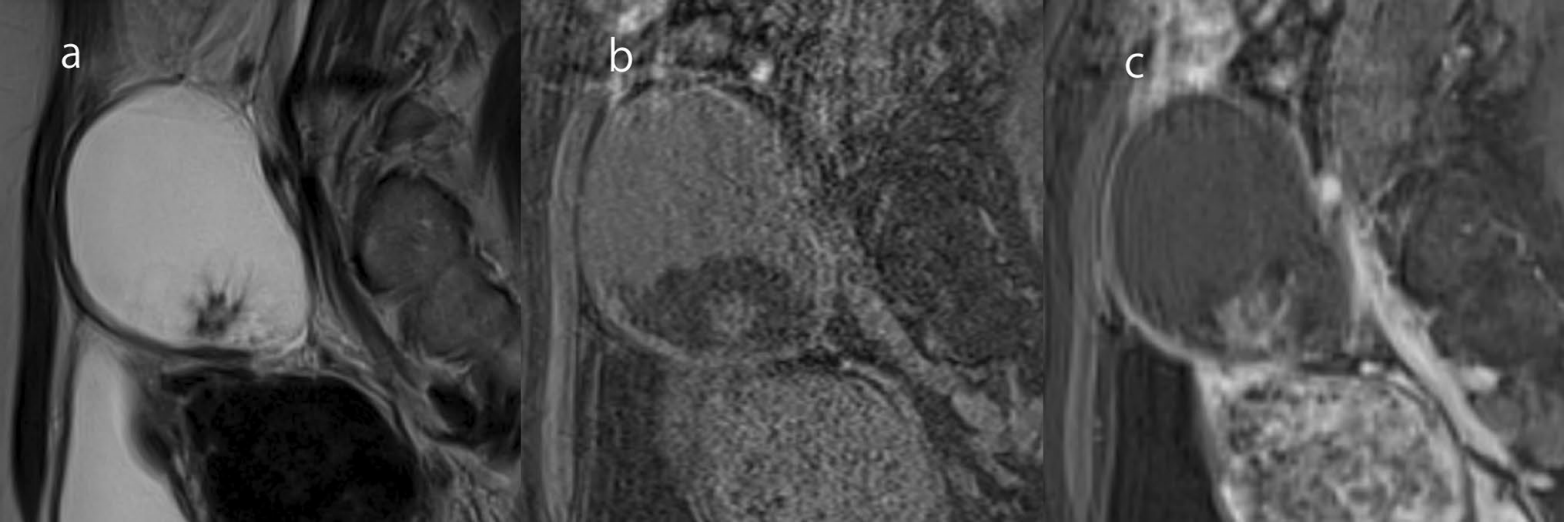
Serous borderline tumor in a woman in her 50 s. **a** A unilocular cystic mass is found at the head of the uterus. The inside of the cyst is markedly hyperintense on T2WI. A papillary mural nodule can be seen rising from the inferior wall into the lumen. The mural nodule has a dendritic hypointense area at its center and a marked hyperintense area around it. This forms the so-called ‘papillary architecture and internal branching pattern.’ The signal intensities of the cyst content and papillary mural nodule margins are similar, and the contour of the mural nodule is, therefore, unclear. **b** On FS-T1WI, the mural nodule margins are hypointense compared with the cyst content, and the contour of the mural nodule is clear. **c** Dendritic enhancement of the mural nodule can be seen on Gd-enhanced FS-T1WI

**Fig. 9:**
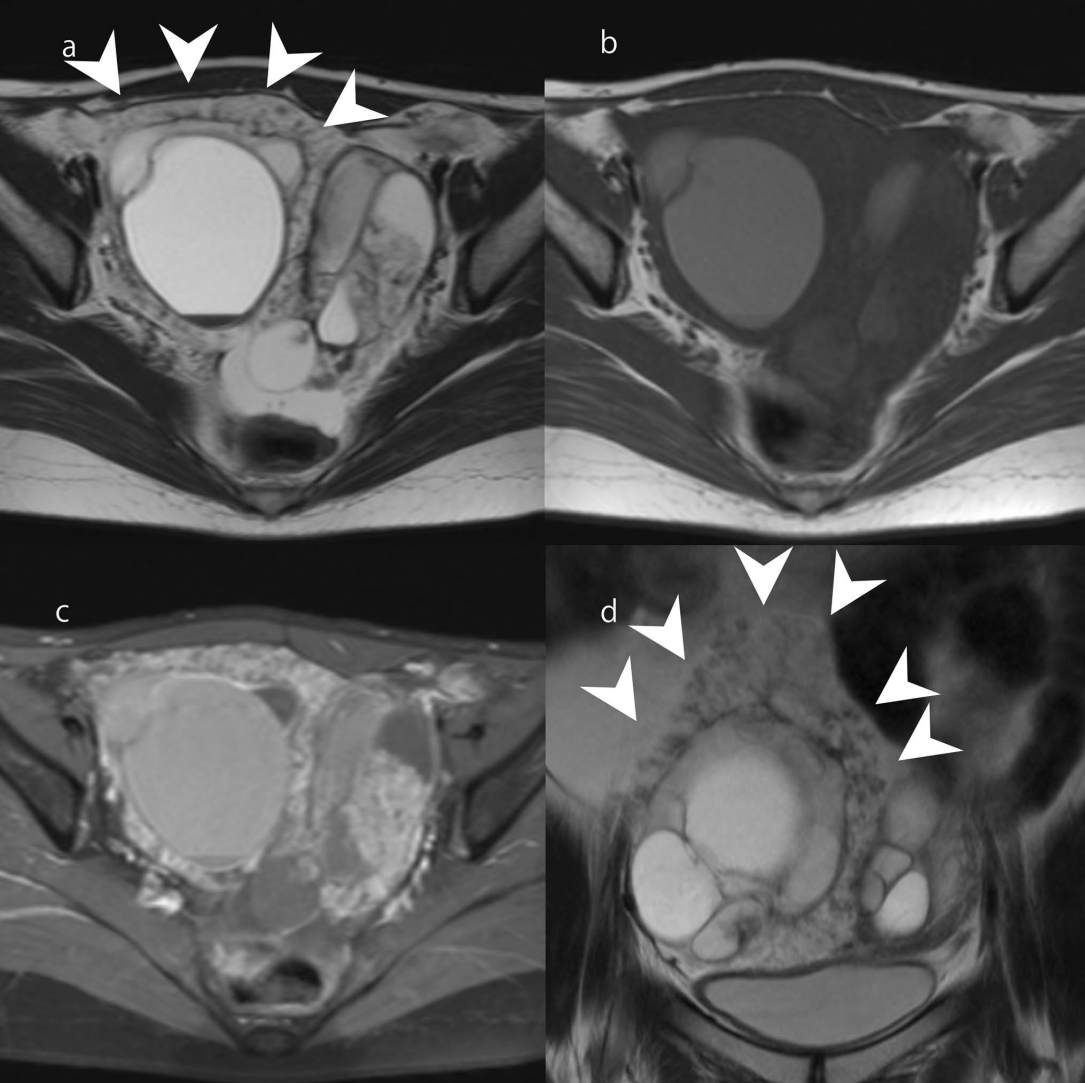
Bilateral serous borderline tumor in a woman in her 20 s. **a** Bilateral ovarian multilocular cystic masses with solid components have formed. The right ovarian mass has exophytic solid components (arrowhead), whereas the left ovarian mass has intracystic solid components. T2WI shows that the solid components of the bilateral ovarian mass are composed of markedly hyperintense areas with dendritic hypointense areas. **b**, **c** The solid components are hypointense on T1WI (**b**) and show strong enhancement on Gd-enhanced T1WI (**c**). The cystic content is hyperintense; hence, the lesion needs to be differentiated from tumors associated with endometriosis. **d** On the coronal T2WI, the exophytic solid components (arrowhead) of the right ovarian mass shows well-depicted papillary architecture and an internal branching pattern

Serous borderline tumor is a non-invasive low-grade serous epithelial neoplasm [1]. It occurs in patients of a wide age range, and 37–55% of cases show bilateral lesions [1, 6]. Macroscopically, it may have intracystic and/or exophytic solid components with surface involvement [1]. One of the characteristic histopathological findings of serous borderline tumor is hierarchical branching papillae with variable amounts of edematous stroma in the core [1, 6]. On MRI, intracystic and/or exophytic papillomatous solid components are observed, reflecting these architectural properties (Figs. 8, 9). On T2WI, the centers of the papillary solid components show dendritic hypointensity reflecting fibrous stroma, and the surrounding area shows marked hyperintensity reflecting edema of the stroma (Figs. 8, 9) [30]. This finding called ‘papillary architecture and internal branching pattern’ is a characteristic finding of serous borderline tumor [30]. The cystic content of serous tumors shows various intensities depending on the protein concentration and the presence of hemorrhage. It should be noted that the cystic contents may occasionally show as hyperintense on fat-suppressed T1WI, and need to be differentiated from neoplastic lesions related to endometriosis (Fig. 9).

### High-grade serous carcinoma

**Fig. 10:**
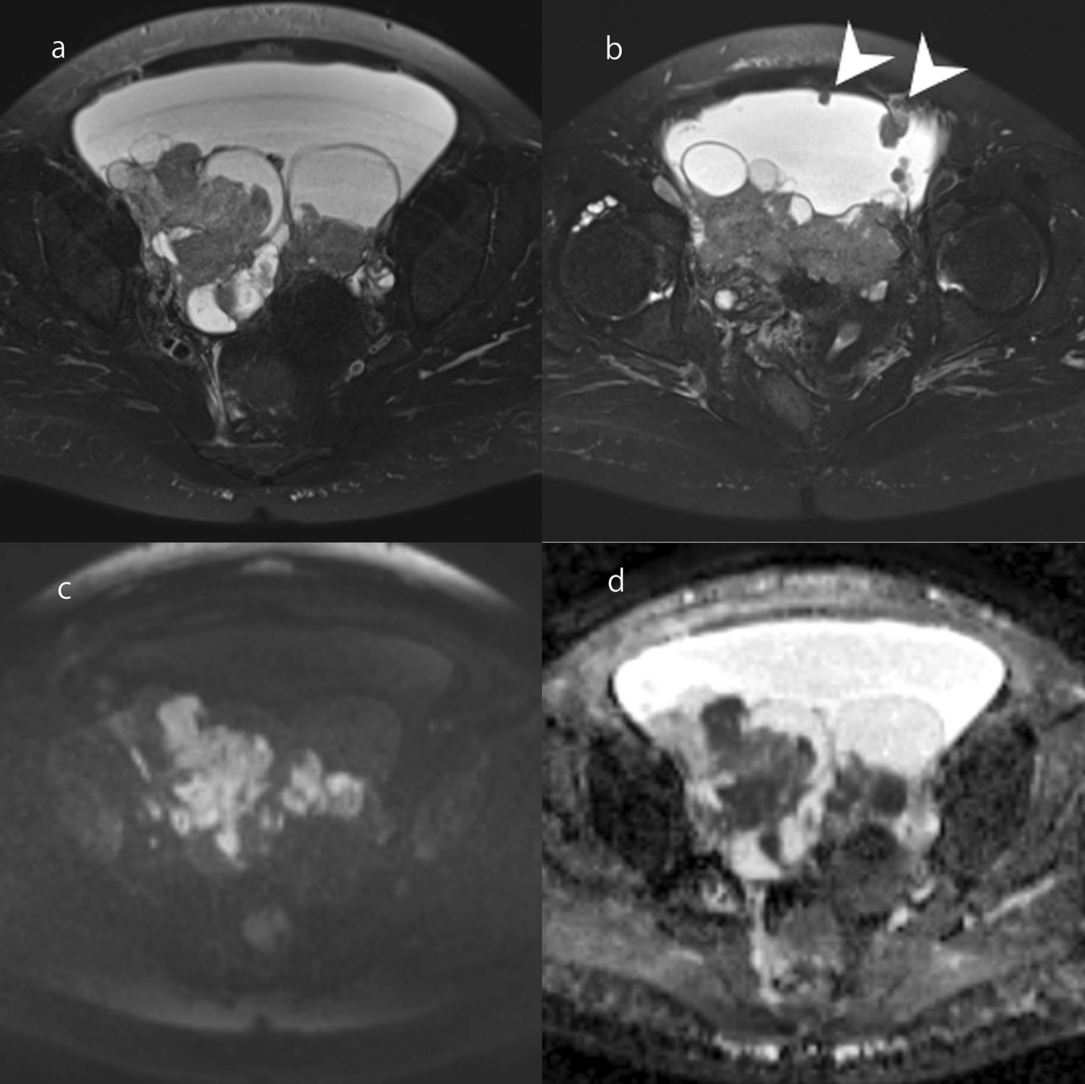
High-grade serous carcinoma. **a** FS-T2WI shows bilateral ovarian solid and cystic masses. Solid components show hypointensity compared with serous borderline tumor. A large amount of ascites is visible. **b** FS-T2WI shows some peritoneal dissemination (arrowhead). **c** The solid components are markedly hyperintense on DWI (high *b* value). **d** The solid components are markedly hypointense on ADC maps

High-grade serous carcinoma is the most common ovarian carcinoma [1, 6]. It is usually bilateral, large, and exophytic, and is composed of papillary solid components and cystic components [1, 6]. Tumors are detected at FIGO stage 3 or 4 in approximately 80% of patients, and extensive extraovarian involvement is commonly observed [1]. The degree of cellular atypia is high, and the stroma of the solid papillary component is narrower than in serous borderline tumor [1, 6]. On MRI, intracystic and/or exophytic papillary solid components are observed, but the intensity of solid components on T2WI is lower than in serous borderline tumor, reflecting its narrow stroma (Fig. 10). Solid components are markedly hyperintense on DWI and hypointense on ADC maps (Fig. 10) [31, 32]. A large amount of ascites and numerous peritoneal disseminations are often observed (Fig. 10) [33]. When the cystic content shows hyperintensity on fat-suppressed T1WI or when the peritoneal dissemination is inconspicuous, it may be necessary to distinguish high-grade serous carcinoma from endometrioid carcinoma and clear cell carcinoma, as described later. Serum carbohydrate antigen 125 is markedly elevated in patients with high-grade serous carcinoma, but it is a non-specific tumor marker [1].

### Cystic tumor with solid component that show as hypointense on T2WI and DWI

**Fig. 11:**
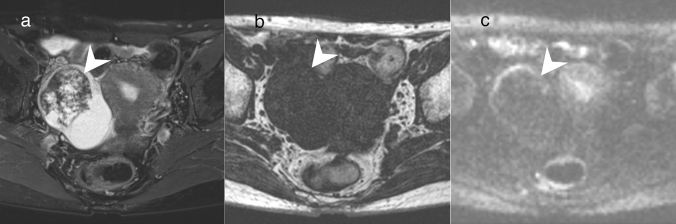
Cystic tumor with solid components showing hypointensity on T2WI and DWI. **a**–**c** Fibroma in a woman in her 40 s. A solid and cystic mass is present in the left ovary. The solid components are hypointense on FS-T2WI, comparable to skeletal muscle (arrowhead) (**a**). The solid components are hypointense on FS-T1WI, comparable to skeletal muscle (arrowhead) (**b**). The solid components are hypointense on high b value DWI (arrowhead) (**c**)

Tumors with solid components showing as a hypointense area on T2WI and DWI correspond to an O-RADS MRI score of 2, indicating that they are very likely to be benign tumors [4]. Such tumors include adenofibroma, benign Brenner tumor, fibroma, and thecoma. With the exception of adenofibroma, these tumors are commonly solid tumors, but occasionally form cystic component-dominated masses. In all of these tumors, the abundance of collagen fibers in the solid components is reflected by their low signal intensity on T2WI (Fig. 11) [34–37]. A black sponge-like appearance has been reported to be a characteristic MRI finding of adenofibroma [34]. Adenofibroma can be an origin of endometrioid carcinoma or clear cell carcinoma, same as endometriosis [1]. In clear cell carcinoma originating from clear cell adenofibroma, a part of the solid component showed hypointense on T2WI [38]. Calcification is often observed in the solid components of benign Brenner tumor, and it can be a clue to the differentiation of this tumor type [36].

## Cystic mass with mural nodule or solid component related to endometriosis

Among the tumors originating from endometriosis, endometrioid carcinoma, clear cell carcinoma, and seromucinous borderline tumor are common, but endometrial stromal sarcoma is rare [2]. Here, we describe mainly the former three. We also describe decidualization of endometriosis and polypoid endometriosis, which need to be differentiated from these tumors.

Before discussing these lesions, attention should be paid to findings suspected to be related to endometriosis. If the cystic content of a tumor shows hyperintensity on fat-suppressed T1WI, a hemorrhagic component within the cyst should be suspected. However, high signal may not only be caused by hemorrhagic components, but also by high concentrations of proteins. Even if the hyperintensity on fat-suppressed T1WI is considered to be bleeding, it is difficult to determine whether it is hemorrhage due to endometriosis or bleeding from tumor. For this reason, serous tumors unrelated to endometriosis may also be listed in the differential diagnosis. If there is suspicion of the tumor being related to endometriosis, it should be checked whether past MRI shows endometriosis or whether the patient has a history of endometriosis. In addition, the presence of adenomyosis and rare-site endometriosis may suggest that ovarian lesions are associated with endometriosis.

### Seromucinous borderline tumor

**Fig. 12:**
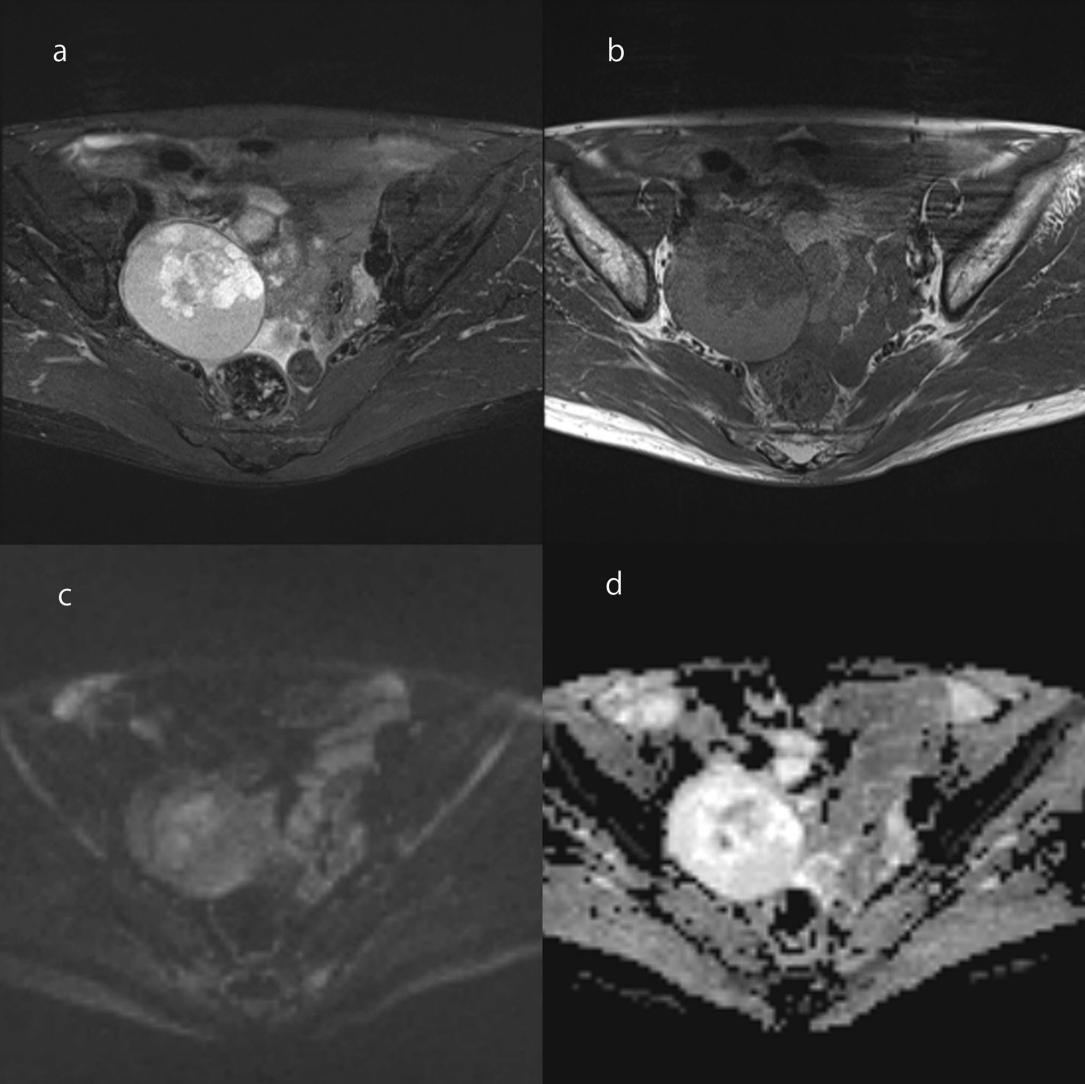
Seromucinous borderline tumor in a woman in her 60 s. **a** FS-T2WI shows the right ovarian cystic mass to have slightly hyperintense content with a papillary mural nodule. The papillary mural nodule shows marked hyperintensity with central dendritic hypo-intensities. This finding resembles those of serous borderline tumor. **b** On T1WI, the right ovarian cystic mass has slightly hyperintense content with a hypointense mural nodule. **c** On DWI (high b value), the central area of the mural nodule is slightly hyperintense. **d** On ADC maps, the central area of the mural nodule is slightly hypointense but the peripheral area of the mural nodule is markedly hyperintense

Seromucinous borderline tumor (formerly called endocervical-type mucinous borderline tumor) is a relatively rare ovarian tumor that originates from endometriosis [1, 6]. One-third of patients with this tumor have endometriotic cysts [1]. Pathologically, seromucinous borderline tumor is composed of papillae exhibiting hierarchical branching with edematous fibrous stroma [1, 6]. The epithelium lining the papillae is an admixture of Mullerian cell types (including endometrioid, ciliated, mucinous, and hobnail types) in varying proportions [1, 6]. The morphologic and behavioral features are shared with serous borderline tumor [6]. MRI findings reflect these pathological characteristics, and it appears as an endometriotic cyst with papillary mural nodules (Fig. 12) [39]. The mural nodules show as dendritic hypointensity with surrounding markedly hyperintense areas on T2WI (Fig. 12) [39, 40]. This reflects the papillary architecture and internal branching pattern, a finding that is useful for differentiating it from endometrioid carcinoma and clear cell carcinoma, which also originate from endometriosis [40]. Although serous borderline tumor may show similar findings to seromucinous borderline tumor, the clues to their differentiation are the strong enhancement of the mural nodule and exophytic growth suggesting serous borderline tumor, and the cystic content with hyperintensity on T1WI and hypointensity on T2WI suggesting seromucinous borderline tumor [39].

### Endometrioid carcinoma

**Fig. 13:**
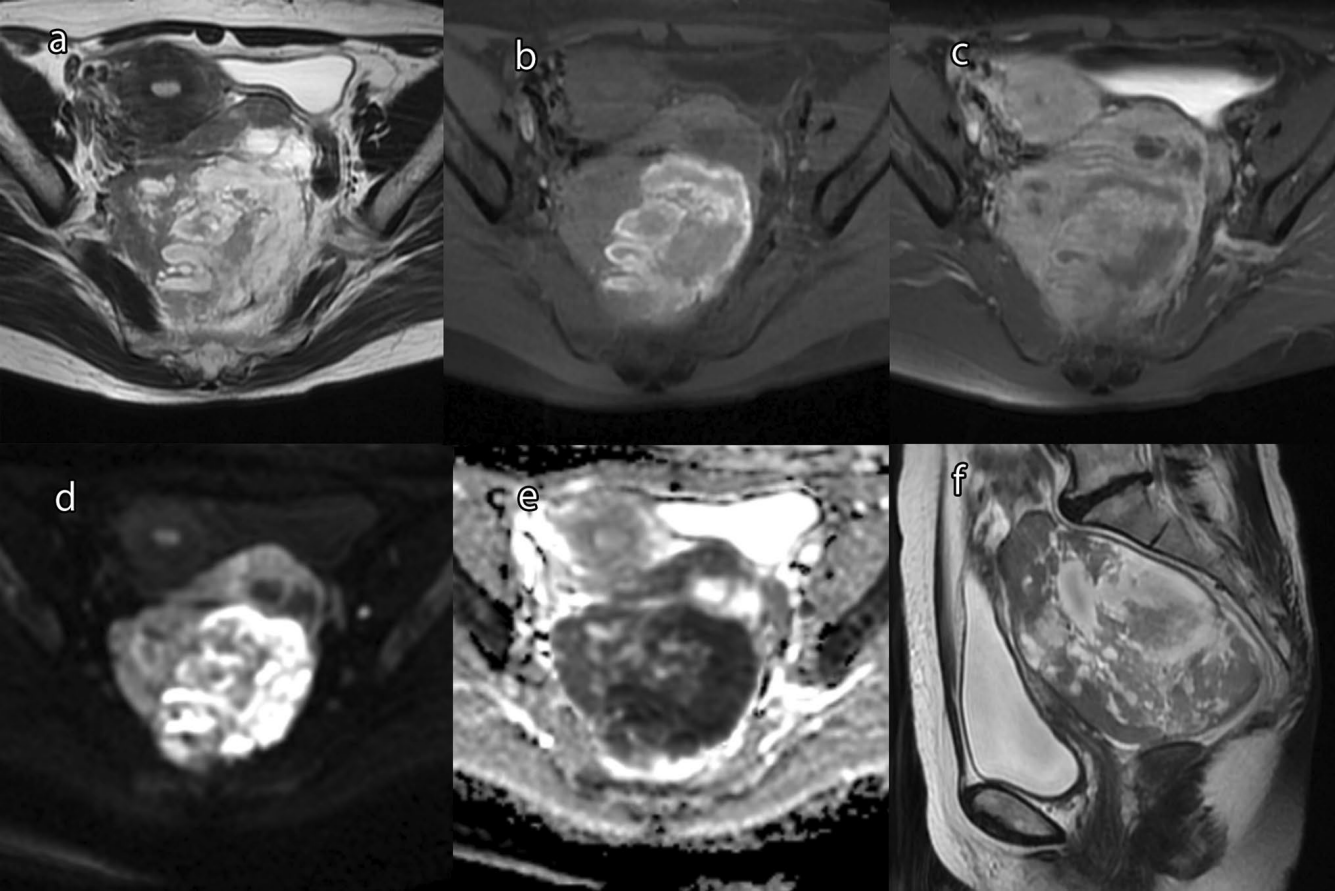
Endometrioid carcinoma in a woman in her 60s. **a** T2WI shows a mass in the left ovary consists of a cyst and solid components. **b** FS-T1WI shows a hyperintense area inside the mass, suggesting hemorrhage. **c** Gd-enhanced FS-T1WI shows that the solid components have the same degree of enhancement as the myometrium. **d** On DWI (high *b* value), the solid components are hyperintense compared with the myometrium. The hemorrhage area shows marked hyperintensity. **e** ADC maps show the solid components to be hypointense compared with the myometrium. **f** Sagittal T2WI shows the solid components to be multicentric

Endometrioid carcinoma accounts for approximately 10% of all ovarian carcinomas [41]. Approximately 90% of endometrioid carcinomas originate from endometriosis [1], whereas a small number tumors originate from benign or borderline adenofibromas [1]. Endometrioid carcinomas are usually unilateral, and are typically composed of solid and cystic components [1]. If originating from endometriotic cyst, a polypoid nodule is formed projecting into the lumen of a blood-filled cyst [1]. On MRI, endometrioid carcinoma typically appears as an endometriotic cyst with a mural nodule. In this case, it is important to note that T2WI rarely shows “shading” in the cystic component [42]. Most of the solid components that show moderate or slight hyperintensity on T2WI are multicentric and concentric (Fig. 13) [42, 43]. In addition, the ADC value of solid components may be lower than in other ovarian malignant tumors [44].

**Fig. 14:**
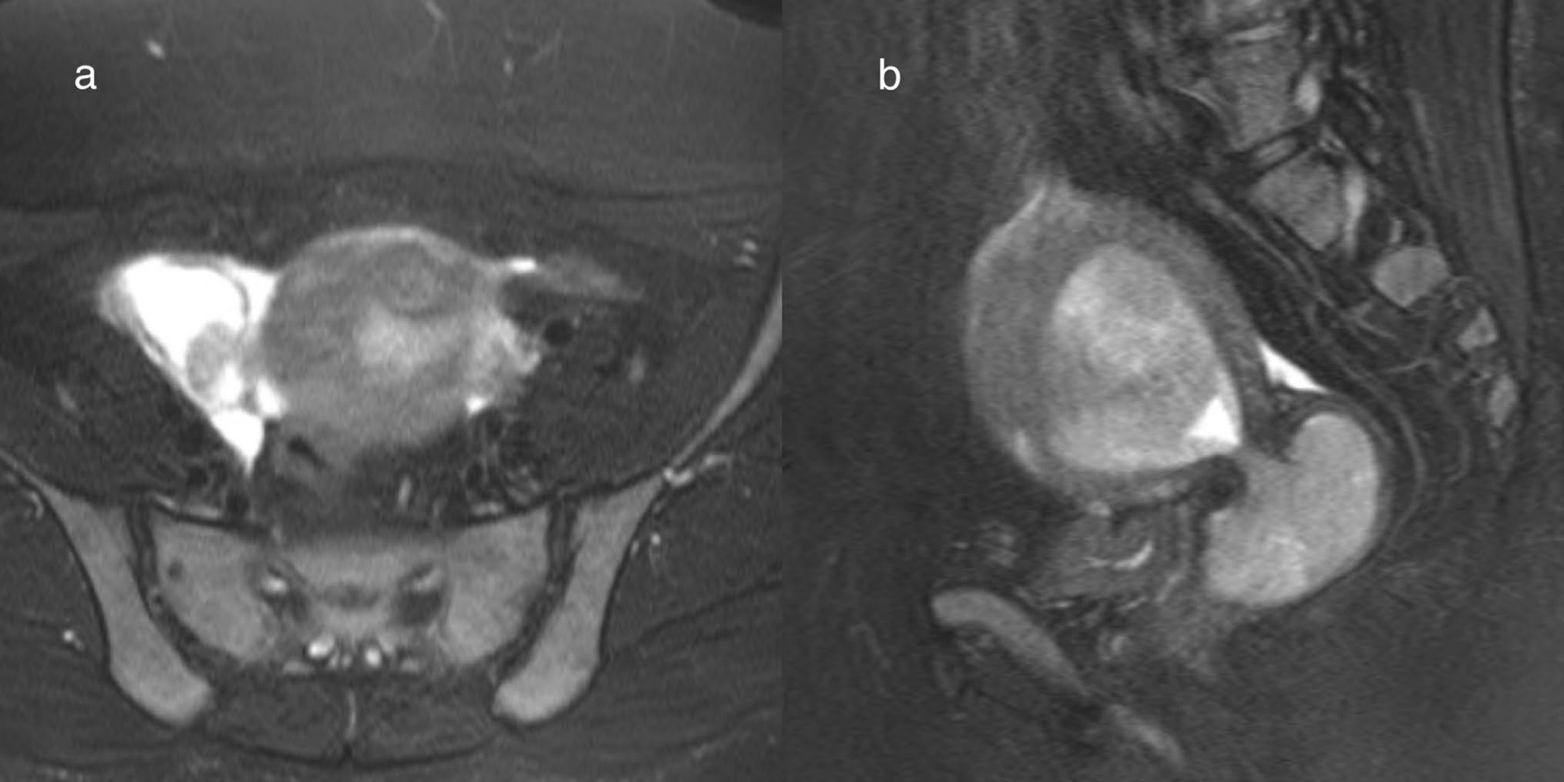
Endometrioid carcinoma of the right ovary with endometrioid carcinoma of the uterine corpus in a woman in her 40s. **a** A cystic mass is present in the right ovary. FS-T2WI shows mural nodules with a moderate signal intensity. One mural nodule is focal and eccentric. This is a finding that is more likely to be associated with clear cell carcinoma. **b** Sagittal FS-T2WI shows that a mass suspected to be endometrial carcinoma is present in the uterine cavity and is hanging down into the cervix. Based on the above, the mass in the right ovary is strongly suspected to be endometrial carcinoma

It is known that approximately 14% of patients with endometrioid ovarian carcinoma have endometrial carcinoma of the uterus [6]. Both tumors are often well-differentiated endometrioid carcinoma [6]. When they coexist, it is extremely difficult to determine which is the primary lesion or metastasis on pathological analysis [6]. However, when an ovarian mass and endometrial lesion are present at the same time, both are likely to be endometroid carcinomas (Fig. 14).

### Clear cell carcinoma

**Fig. 15:**
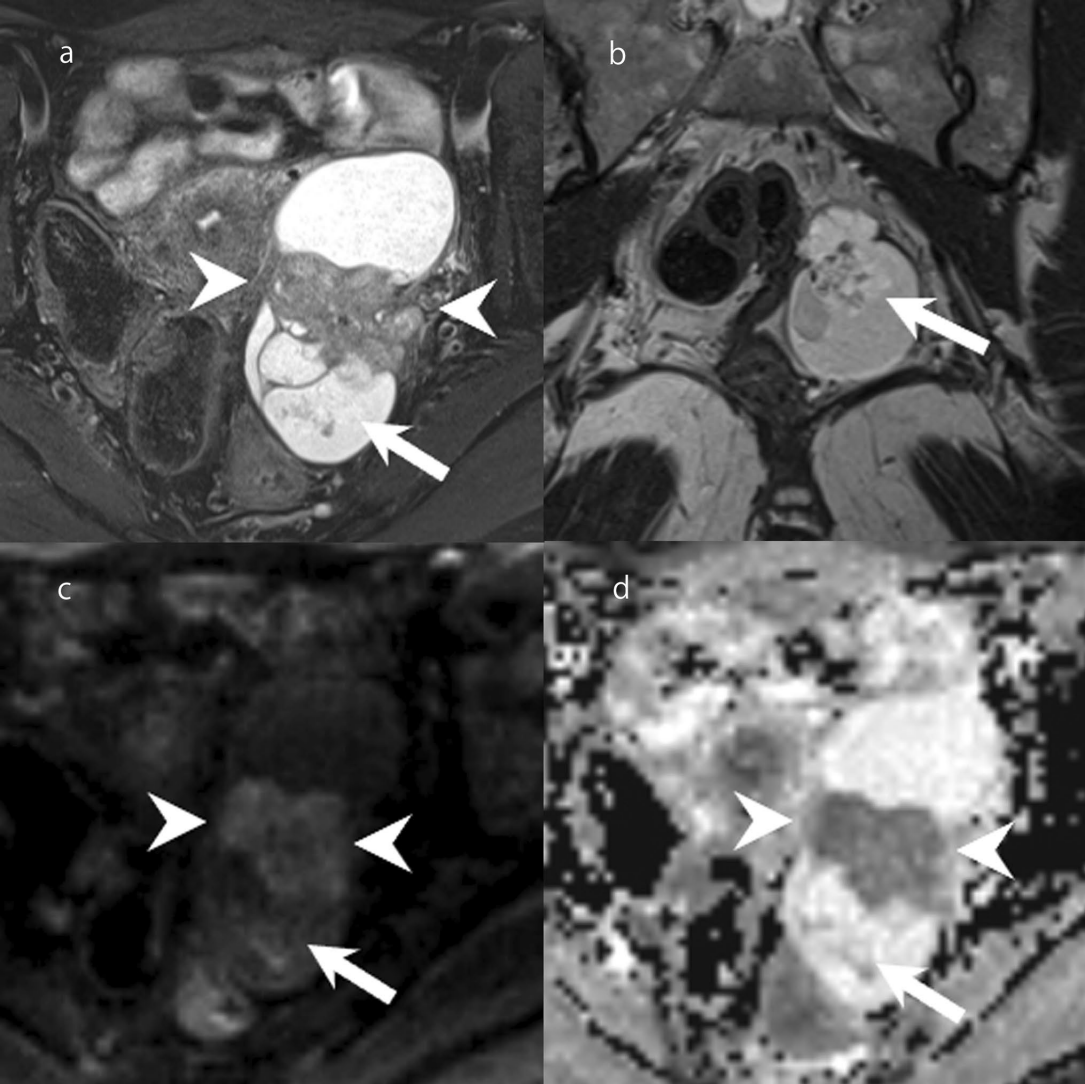
Clear cell carcinoma of a woman in her 40s. **a** A mass consisting of cysts and solid components was found in the left ovary. The center of the mass has a solid component with moderate hyperintensity on FS-T2WI (arrowhead). There is also a papillary mural nodule in the posterior mass loculus that shows a markedly hyperintense area with a dendritic hypointense area in the center on FS-T2WI (arrow). **b** A papillary mural nodule is clearly depicted on coronal T2WI (arrow). **c** On DWI (high *b* value), the solid component in the center of the mass is slightly hyperintense (arrowhead), whereas the papillary mural nodule is hypointense (arrow). **d** On ADC maps, the solid component in the center of the mass is hypointense (arrowhead), whereas the papillary mural nodule is hyperintense (arrow)

Clear cell carcinoma is composed of a mixture of clear, eosinophilic, and hobnail cells [1, 6]. It accounts for 27% of ovarian carcinomas in Japan [45]. Endometriotic cysts are found in 50–74% of patients with clear cell carcinoma [46–48]. Clear cell carcinomas are typically unilateral [1]. The cut surface of the tumor varies from solid, to solid and cystic, to predominantly cystic [1]. If originating from endometriotic cyst, fleshy pale-yellow nodules are formed projecting into the lumen of a blood-filled cyst [1]. Microscopically, clear cell carcinomas have tubulocystic, papillary, and solid architecture, which often occur together [6]. Clear cell carcinomas are divided into those arising from endometriosis forming cysts and those arising from adenofibromas [6]. The former tends to have a papillary pattern and the latter a tubulocystic pattern [6]. On MRI, the findings of mural nodules or solid components are diverse, reflecting the diverse tissue structures (Fig. 15). When presenting with a cystic mass, the mural nodules are commonly focal and eccentric [43]. Mural nodules often appear moderately hyperintense on T2WI, but may also appear markedly hyperintense similar to serous borderline tumors or seromucinous borderline tumors, or hypointense similar to adenofibromas (Fig. 15) [38]. In addition, the ADC values of mural nodules or solid components are higher than those of high-grade serous carcinomas and endometrioid carcinomas (Fig. 15) [49]. It should be noted that there is an overlap between the findings of endometrioid carcinoma and clear cell carcinoma, and it is sometimes difficult to distinguish them. In comparison with high-grade serous carcinoma, which is less associated with endometriosis, clear cell carcinomas are typically unilateral and localized [33]. In addition, the cyst contents of clear cell carcinomas often appear more hyperintense on T1WI, while mural nodules are taller and show less peritoneal dissemination and ascites [33]. Among epithelial ovarian tumors, clear cell carcinoma is most strongly associated with thromboembolism and hypercalcemia [50, 51].

### Polypoid endometriosis

**Fig. 16:**
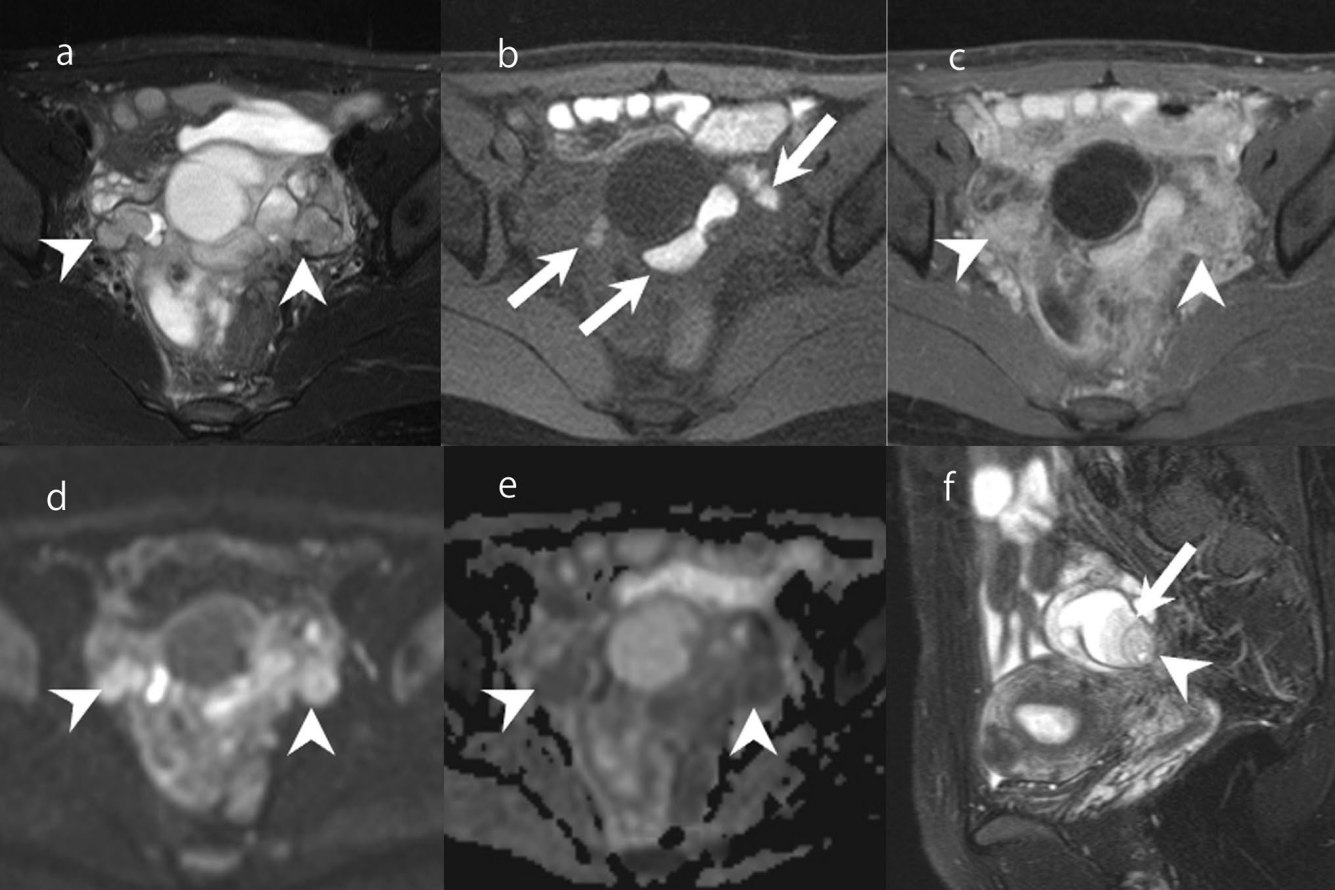
Polypoid endometriosis in a woman in her 30s. **a** A lesion consisting of cystic and solid components is visible in the pelvis. The solid components are moderately hyperintense on FS-T2WI (arrowhead). **b** Some of the cystic components are markedly hyperintense on fat-suppressed FS-T1WI (arrow). **c** Solid components have moderate enhancement on Gd-enhanced FS-T1WI (arrowhead) and are hyperintense on DWI (**d**: arrowhead). **e** On ADC maps, the solid components are iso-intense compared with skeletal muscle (arrowhead). **f** Sagittal FS-T2WI shows the solid component as iso-intense compared with endometrium (arrowhead), and there is a hypointense rim on the surface of the solid component (arrow)

In addition to a cystic appearance, endometriotic lesions may also appear as nodular lesions [2, 52]. Rarely, endometriosis forms papillary masses that protrude from the serosal surfaces into the lumens of endometriotic cysts [52]. There are very few studies of polypoid endometriosis including a large number of patients, but there are more than 100 case reports. In some cases, there is history of exogeneous estrogen use [52]. The morphology of the lesion is clinically similar to that of malignant tumor [52]. Polypoid endometriosis shows similar MRI findings to endometriosis, with lesions that are hyperintense on T2WI and hypointense on T1WI (Fig. 16) [53]. The lesions show hyperintensity on DWI and a slight hypointensity on ADC maps (Fig. 16) [54]. Occasionally, a hypointense rim reflecting fibrous tissues on the surface of the solid components is seen on T2WI. This finding is useful for differentiating polypoid endometriosis from malignant tumors such as clear cell carcinoma and endometrioid carcinoma (Fig. 16) [53, 55].

### Decidualized endometriosis

**Fig. 17:**
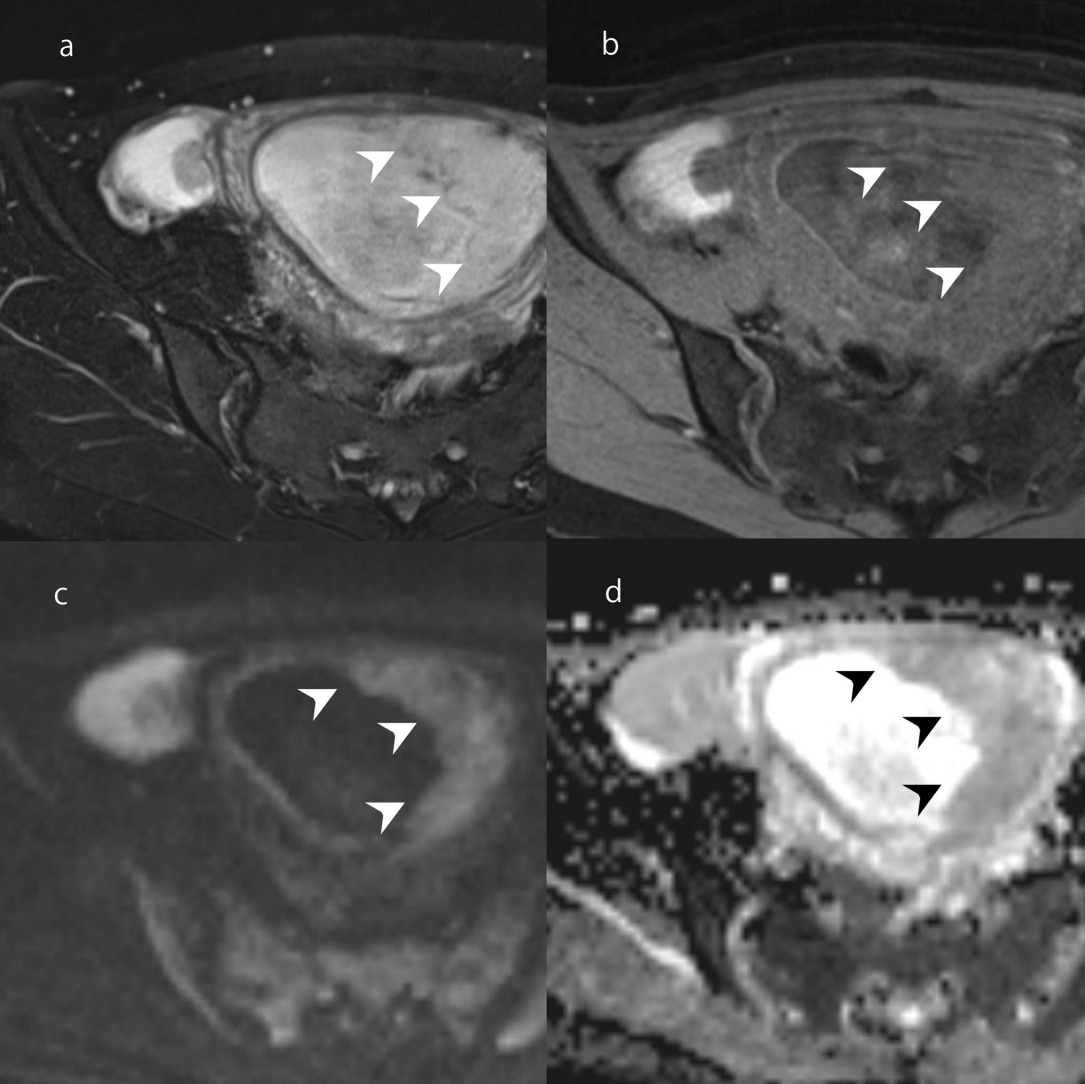
Decidualized endometriosis in a woman in her 20s at 14 weeks of pregnancy. **a** A cystic mass with a mural nodule is observable in the right ovary. On FS-T2WI, the mural nodule is as hyperintense as the placenta (arrowhead). **b** On FS-T1WI, the mural nodule is iso-intense compared with the placenta (arrowhead). **c** On DWI, the mural nodule is as hyperintense as the placenta (arrowhead). **d** On ADC maps, the mural nodule is as hypointense as the placenta (arrowhead)

Decidualized endometriosis is one of the changes in the form of endometriosis that may occur during pregnancy [56]. Decidual changes of ectopic endometrial tissues in endometriotic cyst sometimes form mural nodules and mimic malignant transformation [57–59]. On MRI, the mural nodules show marked hyperintensity on T2WI and hypointensity on T1WI (Fig. 17) [57–59]. The mural nodules of decidualized endometriosis are hyperintense on DWI and ADC maps because of a T2-shine through effect (Fig. 17) [59]. However, mural nodules or solid components of malignant transformations generally show hyperintensity on DWI and low ADC values. If decidualized endometriosis is suspected, follow-up examinations using ultrasound are required. If the lesion is decidualized endometriosis, its size will decrease over time.

## A cystic mass with fat or liquid contents

Findings of fat or lipid contents in a cystic mass almost confirm that the mass is a teratoma-related lesion because pure lipomas and liposarcoma are extremely rare in the ovaries [60]. Lesions with lipid contents and without solid components are given a score of 2 in the O-RADS MRI risk stratification system and are diagnosed as benign lesions [4], whereas lesions with lipid contents and a large volume of enhancing soft tissues are given a score of 4 [4].

### Mature cystic teratoma

**Fig. 18:**
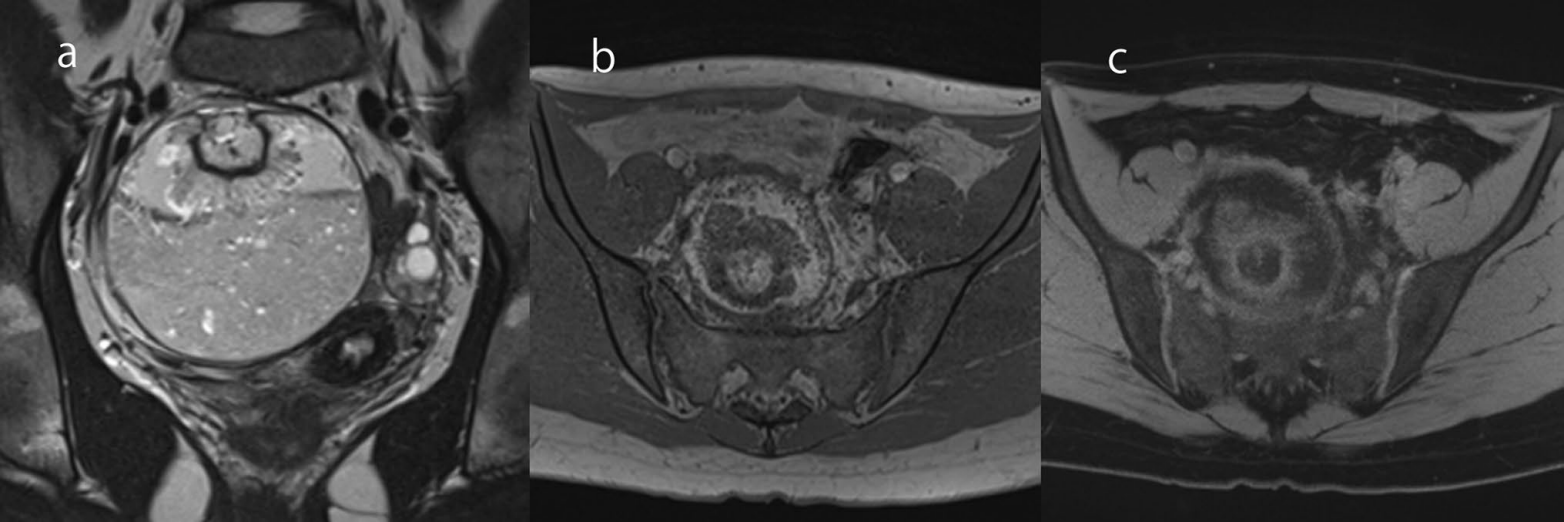
Mature cystic teratoma in a woman in her 20s. **a** A coronal T2WI shows an 8-cm cystic mass in the right ovary with a protruding structure with a palm tree appearance. **b** The cystic content and central area of the protruding structure are hyperintense on T1WI and hypointense on FS-T1WI (**c**). From the above, it can be seen that there is fat inside the protruding structure, and that it is a Rokitansky protuberance

Mature teratoma is a tumor composed of mature tissue originated from two or three germ layers [1]. This tumor accounts for 20% of all ovarian neoplasms [1] and is commonly seen as a cystic lesion [1]. Sebaceous materials, hair, and teeth of cartilage may be included in the lesion. On MRI, the fat-containing cystic contents are hyperintense on T1WI and T2WI, with the signal being markedly lower on fat-suppressed imaging. A ball-shaped object called a floating fat ball may float inside the cyst. Projections called Rokitansky protuberances, which are lined with hair-bearing skin, are often found [1]. On MRI, Rokitansky protuberance is seen as a mural nodule-like structure with internal fat and calcification (Fig. 18).

### Malignant transformation of mature cystic teratoma

**Fig. 19:**
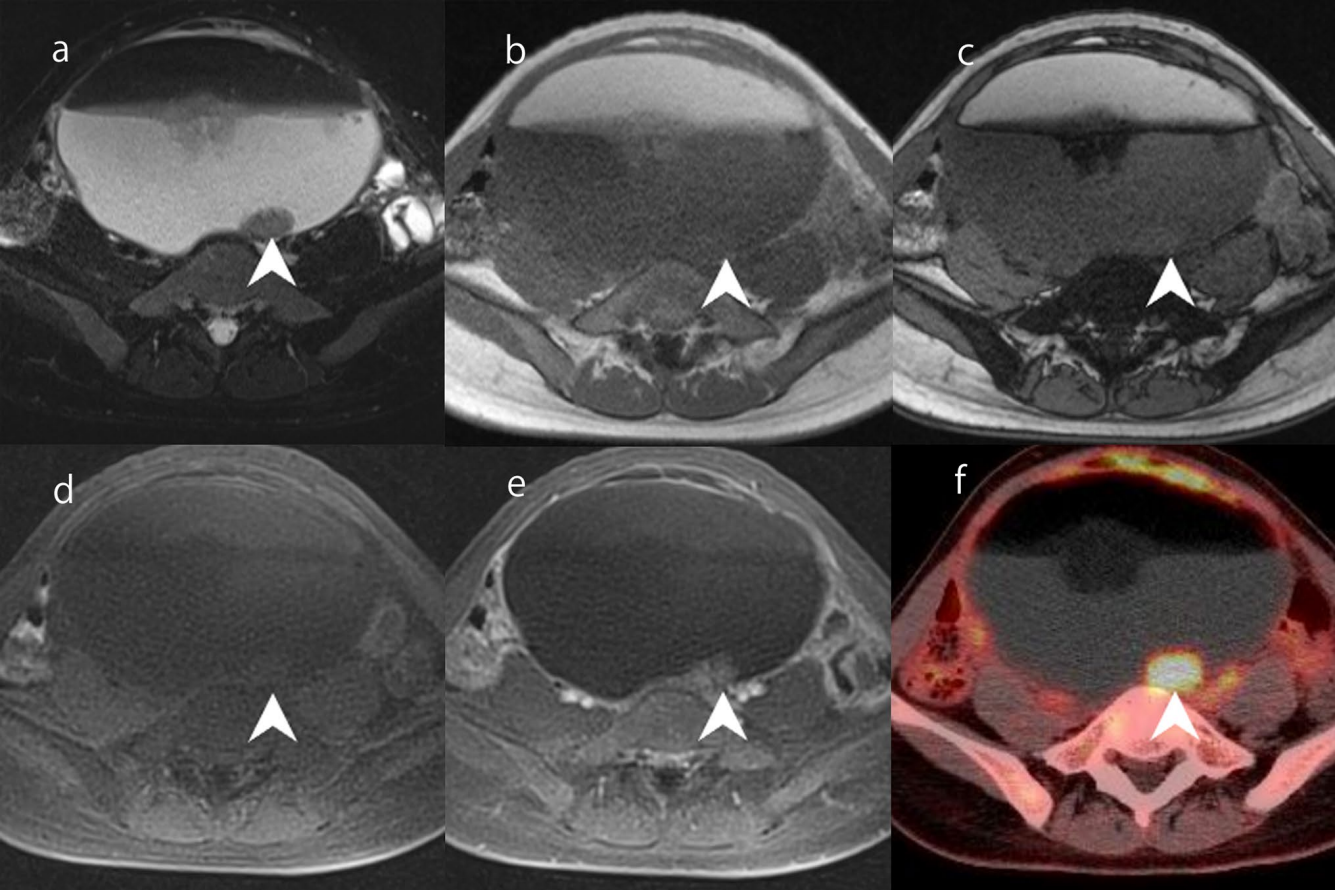
Malignant transformation of mature cystic teratoma in a woman in her 20 s. **a** FS-T2WI shows a cystic mass with an internal fluid–fluid level. A moderately hyperintense 2-cm mural nodule on the posterior wall of the mass (arrowhead) is observable on FS-T2WI. T1-weighted gradient-echo in-phase (**b**) and opposed-phase (**c**) imaging shows that the mural nodule has no fat or lipid content (arrowhead). **d** FS-T1WI shows lipid-rich fluid and a hypointense mural nodule (arrowhead). **e** Gd-enhanced FS-T1WI shows mild enhancement of the mural nodule (arrowhead). **f** 18F-FDG-PET/CT shows significant accumulation in the mural nodule (arrowhead). Surgery was performed and malignant transformation (squamous cell carcinoma) of mature cystic teratoma was diagnosed

Malignant transformation is a rare complication of mature cystic teratoma [61]. It can occur in a wide range of age groups, but is usually observed in postmenopausal patients [62]. Various types of malignant tumors can develop from malignant transformation of any tissue in mature teratoma. The most common histological type is squamous cell carcinoma. In some reports [63, 64], malignant transformation occurred in mature cystic teratomas larger than 10 cm. Unlike common ovarian carcinoma, it is characterized by transmural extension and infiltration into surrounding organs from solid components [65]. Unlike Rokitansky protuberances, the solid components of malignant transformation do not usually have fat or calcification inside (Fig. 19). In addition, diffusion restriction on DWI and strong accumulation on FDG-PET are observed in such mural nodules (Fig. 19). If an elderly woman has a teratoma larger than 10 cm, attention should be paid to the possible presence of mural nodules without fat or calcification. In O-RADS, the presence of a large volume of contrast-enhancing solid components in a lesion with lipid content presents an intermediate risk [4]. However, it should be noted that even small mural nodules can be squamous cell carcinoma.

### Mucinous tumors arising in mature cystic teratoma

Most primary ovarian mucinous tumors originate from surface epithelial inclusion cysts [6]. However, a small number of primary ovarian mucinous tumors arise from germ cells, and 2–11% of ovarian mature cystic teratomas are associated with mucinous tumors [6]. These are histologically similar to adenomatous tumors of the lower gastrointestinal tract, and are associated with pseudomyxoma ovarii [6]. Radiologically, mucinous tumors arising from mature cystic teratomas show a multilocular cystic mass with lipid contents, indicating teratomas. If a solid component is found, mucinous carcinoma should be suspected, but it is difficult to distinguish it from malignant transformation of mature cystic teratoma.

## Conclusions

The differential diagnosis of ovarian cystic masses has been described in terms of five types of imaging findings, focusing on the common diseases that are likely to be encountered in daily practice. The differential diagnosis of unilocular cystic masses without mural nodules includes only benign lesions. The most common type of multilocular cystic mass is the mucinous tumor, but it should be noted that the solid components may not be visible, even in borderline or malignant cases. If mural nodules are present in cystic mass, the signal intensity of the mural nodules on T2WI may be helpful in their differentiation. Among tumors of endometriotic cyst origin, seromucinous tumors have relatively characteristic MRI findings. Endometrioid carcinoma and clear cell carcinoma have some similar MRI findings, but secondary findings may be helpful for differentiating them. In teratoma-related lesions, the size, patient age, and mural nodule characteristics should be noted. Differentiating ovarian tumors is difficult. However, estimation of not only whether the tumor is benign or malignant but also the histological type provides better treatment options.
